# Microbial and Isotopic Evidence for Methane Cycling in Hydrocarbon-Containing Groundwater from the Pennsylvania Region

**DOI:** 10.3389/fmicb.2017.00593

**Published:** 2017-04-05

**Authors:** Adrien Vigneron, Andrew Bishop, Eric B. Alsop, Kellie Hull, Ileana Rhodes, Robert Hendricks, Ian M. Head, Nicolas Tsesmetzis

**Affiliations:** ^1^School of Civil Engineering and Geosciences, Newcastle UniversityNewcastle upon Tyne, UK; ^2^Biodomain, Shell International Exploration and Production Inc.Houston, TX, USA; ^3^DOE Joint Genome InstituteWalnut Creek, CA, USA; ^4^Shell Global Solutions US Inc.Houston, TX, USA; ^5^Shell Exploration and Production CompanyHouston, TX, USA

**Keywords:** aquifer, methanogenesis, methanotrophs, ANME, nitrate, Hydrocarbon seeps, NGS

## Abstract

The Pennsylvania region hosts numerous oil and gas reservoirs and the presence of hydrocarbons in groundwater has been locally observed. However, these methane-containing freshwater ecosystems remain poorly explored despite their potential importance in the carbon cycle. Methane isotope analysis and analysis of low molecular weight hydrocarbon gases from 18 water wells indicated that active methane cycling may be occurring in methane-containing groundwater from the Pennsylvania region. Consistent with this observation, multigenic qPCR and gene sequencing (16S rRNA genes, *mcrA*, and *pmoA* genes) indicated abundant populations of methanogens, ANME-2d (average of 1.54 × 10^4^ *mcrA* gene per milliliter of water) and bacteria associated with methane oxidation (NC10, aerobic methanotrophs, methylotrophs; average of 2.52 × 10^3^ *pmoA* gene per milliliter of water). Methane cycling therefore likely represents an important process in these hydrocarbon-containing aquifers. The microbial taxa and functional genes identified and geochemical data suggested that (i) methane present is at least in part due to methanogens identified *in situ*; (ii) Potential for aerobic and anaerobic methane oxidation is important in groundwater with the presence of lineages associated with both anaerobic an aerobic methanotrophy; (iii) the dominant methane oxidation process (aerobic or anaerobic) can vary according to prevailing conditions (oxic or anoxic) in the aquifers; (iv) the methane cycle is closely associated with the nitrogen cycle in groundwater methane seeps with methane and/or methanol oxidation coupled to denitrification or nitrate and nitrite reduction.

## Introduction

Groundwater and aquifers are complex and fluctuating ecosystems of critical importance for geochemical cycles (Griebler and Lueders, 2009), connecting subsurface and surface biomes. These environments are extremely sensitive to perturbations and pollution (Datry et al., 2004). Therefore, presence of methane as well as trace concentrations of other alkanes such as ethane and propane in groundwater is predicted to have an effect on the groundwater ecosystem. Indeed, these carbon substrates can potentially support an important microbial food chain in aquifers (Barker and Fritz, 1981). Previous studies on petroleum hydrocarbon-contaminated aquifers demonstrated changes in microbial community composition following hydrocarbon inputs and the potential for hydrocarbon biodegradation by different microbial communities which were dictated by electron acceptor availability (Vroblesky and Chapelle, 1994; Chapelle et al., 2002). Methane is a common trace constituent of groundwater (Zhang et al., 1998), occasionally representing more than 20% of the total carbon (Barker and Fritz, 1981).

Methane in aquifers may have different origins. Methane of thermogenic origin can rise from deep, as well as shallower hydrocarbon reservoirs into shallower sediment layers and aquifers due to natural gas migration. Thermogenic methane is typically characterized by heavy carbon (−50 to −20‰) and deuterium (−275 to −100‰) isotopic signatures as an effect of the maturation of the organic matter that is the source of thermogenic methane (Whiticar, 1999). Methane can also be produced locally by microbial activities in shallow anoxic aquifers (Beeman and Suflita, 1990; Kleikemper et al., 2005). Biogenic methane is generally characterized by lighter carbon (−110 to −50‰) and deuterium (−400 to −150‰) isotopic signatures depending on the carbon substrate for methanogenesis (Whiticar, 1999).

Biological methane production is only carried out by specific archaea (Jones et al., 1987). Seven lineages of methanogenic archaea are known: Methanosarcinales, Methanocaellales Methanobacteriales, Methanococcales, Methanomicrobiales, Methanopyrales (Luton et al., 2002), and Methanomassiliicoccales (Borrel et al., 2014). Additional uncultured lineages of methanogenic archaea may also exist and members of the *Bathyarchaeota* phylum have been proposed to be methanogens based on metagenome mining (Evans et al., 2015). Although, different carbon sources might be used by these methanogens (Oremland and Polcin, 1982), the final enzymatic reaction leading to methane production is always carried out by methyl co-enzyme M reductase. The *mcrA* gene which encodes the alpha subunit methyl co-enzyme M reductase of therefore represents a marker gene for methane cycling archaea (Luton et al., 2002).

Natural-gradient tracer tests and radiotracers monitoring have indicated that methane oxidation can also occur in groundwater (Smith et al., 1991; Hansen, 1998). Although, specific bacteria such as *Candidatus Methylomirabilis oxyfera* and other members of the candidate division NC10 have been found to oxidize methane in anoxic environment via oxygen formation from nitrogen oxides (Ettwig et al., 2008, 2010), the bio-attenuation of methane via anaerobic methane oxidation is also carried out by lineages of methanotrophic archaea (ANME-1, -2a/b/c/d, and ANME-3). These methanotrophic archaea are taxonomically related to methanogens and frequently detected in anoxic and methane-rich environments such as hydrocarbon seeps (Boetius et al., 2000; Knittel et al., 2005). This process is considered as a natural biofilter against methane emissions, oxidizing a large fraction of the methane produced in marine environments (Knittel and Boetius, 2009). During methane oxidation, lighter methane is oxidized first leading to an enrichment of residual methane with heavier isotopes (Whiticar, 1999). Methane oxidation may be coupled to the reduction of various electron acceptors such as nitrate/nitrite (Raghoebarsing et al., 2006), iron and manganese (Beal et al., 2009). However, in marine sediments and freshwater wetlands, sulfate is the most important electron acceptor (Knittel and Boetius, 2009; Segarra et al., 2015). In these environments ANME archaea and their bacterial partners form dense microbial consortia. The syntrophic character of this process as well as the exact mechanisms of anaerobic methane oxidation remain a matter of debate and may differ according to the archaeal lineage (Lloyd et al., 2011; Milucka et al., 2012; Vigneron et al., 2014a). In a number of cases there is evidence that methane is oxidized to CO_2_ using a reverse methanogenesis pathway which also involves methyl co-enzyme M reductase (Thauer et al., 2008). Therefore, the microbial, and more particularly the archaeal community present in an aquifer may represent an important factor dictating the fate of methane in hydrocarbon-containing anoxic groundwater environments. However, previous molecular investigations of microbial communities in methane-containing aquifers have generally identified aerobic bacterial methanotrophs (Newby et al., 2004; Erwin et al., 2005).

In contrast to anaerobic methane oxidation, aerobic methane oxidation appears to be exclusively carried out by bacteria. Known aerobic methanotrophs are mainly from the phylum *Proteobacteria* and can be classically divided into two assemblages: type I and type II, based on structural characteristics as well as their phylogeny (Lüke and Frenzel, 2011). Type I methanotrophs (Type Ia, Ib, and Ic, previously described as type X) are affiliated to the family *Methylococcaceae* within the *Gammaproteobacteria* whereas Type II methanotrophs from families *Methylocystaceae* and *Beijerinckiaceae* fall within the *Alphaproteobacteria* (Dunfield et al., 1999; Bowman, 2006). However, the diversity of aerobic methanotrophs appears to have been previously underestimated and additional bacterial methanotrophs from the phylum *Verrucomicrobia* have been identified (Dunfield et al., 2007; Sharp et al., 2014). In aerobic methanotrophs, methane is oxidized via methanol, formaldehyde, and formate to carbon dioxide. This process is initiated by the key enzyme, methane monooxygenase. Different variants of methane monooxygenase occur depending on the bacterial lineage: the soluble, cytoplasmic methane monooxygenase (sMMO), and the membrane-bound particulate methane monooxygenase (pMMO). Nearly all aerobic methanotrophs possess a particulate methane monooxygenase while the distribution of sMMO is more limited (Hainbuch, 2015). Therefore, *pmoA* genes, encoding for the alpha-subunit of the particulate methane monooxygenase are considered as a marker for the detection of aerobic methanotrophs.

Natural hydrocarbon and gas seepages are common in the Pennsylvania region and occurrence of methane in water wells has been observed for centuries (Molofsky et al., 2011, 2016). Therefore, this study aimed to investigate methane cycling and related microbial communities in methane-containing aquifers of the Pennsylvania region (Tioga County), from methane origin to methane oxidation. The potential for aerobic and anaerobic oxidation of methane, which remains poorly explored in groundwater ecosystem, was a particular focus of the work. Indeed, although anaerobic methane oxidation has been observed in freshwater wetlands (Segarra et al., 2015), lake sediments (Schubert et al., 2011) and freshwater gas sources (Timmers et al., 2016), knowledge of anaerobic methane oxidation in methane seeps is largely based on marine environments. Anaerobic methane oxidation in groundwater environments has been investigated to a much lesser degree (Flynn et al., 2013), and the focus has been primarily aerobic methane oxidation (Erwin et al., 2005). To characterize the structure and metabolic functions of the microbial communities in methane-containing water wells from the Pennsylvania region (Tioga County), phylogenetic, functional, and quantitative analyses of archaeal and bacterial communities and particularly those involved in methane cycling were undertaken and related to geochemical data from the aquifers.

## Experimental procedures

### Site description and geochemical analyses

A total of 18 samples from different water wells drawing the uppermost aquifers in the Pennsylvania region (Tioga County) were analyzed. The wells were within a 13 km radius within the Tioga River Valley and did not exceed 130 m in depth (Supplementary Figure 1). The hydrogeological setting of the region has been described in detail previously (Williams et al., 1998; Breen et al., 2007). The wells are supplied by unconsolidated aquifers of outwash sand and gravel of Quaternary age or are in communication with bedrock aquifers. Although, the exact hydrogeological features of the private water wells sampled in this study are not known by the well owners, localized recharge and discharge has been observed. The localized nature of the recharge and discharge patterns suggests that the wells are fed from a complex hydrological network and that individual wells are within separate shallow hydrogeological systems.

**Figure 1:**
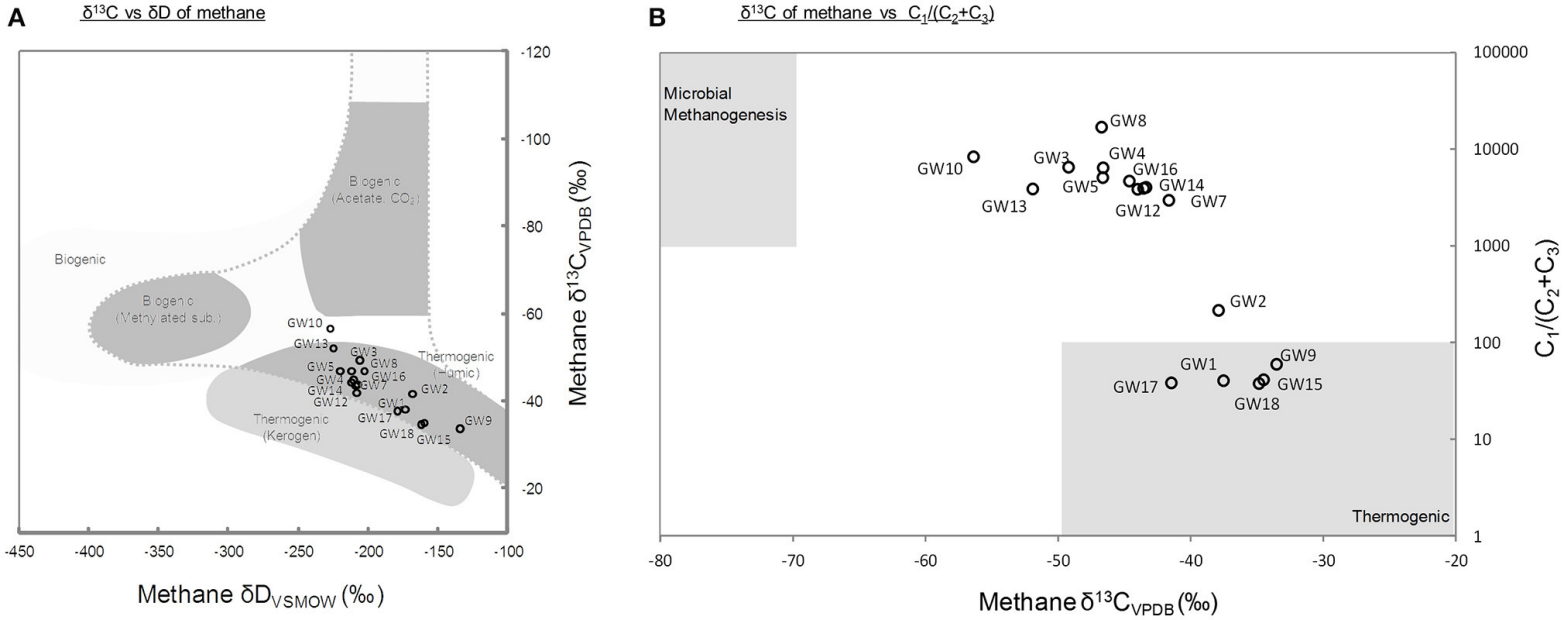
**(A)** Carbon and hydrogen isotopic compositions of methane at the time of the microbial sampling. Gray areas represent typical values for the different methane origins (modified from Whiticar, 1999). **(B)** Ratio of methane to higher-chain hydrocarbons vs. the δ^13^C of methane. Higher-chain hydrocarbon concentrations are historical data measured at the time described in Supplementary Table 2. Gray areas represent typical ranges of thermogenic and biogenic methane (Osborn and McIntosh, 2010). Thermogenic (Kerogen) refers to methane generated from geothermal and hydrothermal alteration of mature organic matter, and thermogenic (Humic) refers to methane generated from thermal alteration of low maturity organic matter.

Known natural gas and shale sources in the area are buried at depths of about 1,190 m below the surface (Breen et al., 2007). Geochemical characteristics of the water from three wells were measured by Isotech laboratories (Champaign, IL, USA) from 2011 to 2014, providing an indication of the degree of fluctuation in methane and oxygen concentrations in the water wells with time (Supplementary Figures 2, 3). Chemical characterization of the water wells was carried out from April 2011 to September 2014 (Supplementary Figure 3). These data were used to provide background information about the environmental conditions that occurred in the water wells, but were not used in analyses that integrated geochemical and microbiological data since the microbiological data were only determined for samples obtained in 2015 (Supplementary Figure 3). Water samples were collected using IsoFlask samplers (Isotech laboratories, IL, USA) during this period (Supplementary Figure 3) and analyzed upon receipt of the samples. Methane concentration and isotopic compositions were also determined in samples taken in 2015. Sampling for microbial community analysis was conducted in 2015, at the same time as sampling for methane and isotope analysis (Supplementary Figure 3, Figure 1). Anions were quantified using a Dionex ICS-2000 ion chromatograph (Thermo scientific, Sunnyvale, CA, USA) with suppressed conductivity detection. Anion were separated on an AS11 column (Dionex) using a KOH gradient. Dissolved gas and stable isotope composition of methane were determined in the headspace of IsoFlask samplers using a Shimadzu GC-2010 gas chromatography system (Shimadzu, Kyoto, Japan) coupled to an isotope ratio mass spectrometry (IRMS) by Isotech laboratories. Procedure for gas analyses were detailed previously (Osborn and McIntosh, 2010; Darrah et al., 2015). Following chromatographic separation, dissolved gas concentrations and isotope compositions were measured by combustion and dual-inlet isotope ratio mass spectrometry (detection limits for C1, C2, and C3 were 0.001, 0.0005, and 0.0001 mol%, respectively).

### Sample collection and DNA extraction

For microbial community analysis, water samples were collected as close as possible to the water well (pressure tank or directly at connected sink) between January and March 2015. Methane concentration and isotopic composition were measured in samples taken at the same time. Consistent with the potential presence of gas in aquifers, effervesce was reported in all water samples. Prior to sampling, each sampling line was flushed for 30 min. An average of 470 ml of water sample was filtered using 0.22 μm Sterivex filters (EMD Millipore, Darmstadt, Germany). Nucleic acids were preserved by addition of 5 ml of RNAlater® and filters were shipped to the laboratory at 4°C for microbial community analysis. On receipt of the samples, DNA was extracted using a PowerLyzer PowerSoil DNA Isolation Kit (Mo Bio, Carlsbad, CA, USA) or PowerWater DNA Isolation Kit (Mo Bio) according to the manufacturer's recommendations and dissolved in autoclaved MilliQ water (Supplementary Table 1). DNA was stored at −20°C prior to analysis. There were no significant differences in microbial community diversity indices, community composition, or abundance between the two DNA extraction methods (Student *T*-test, *P* > 0.5, Supplementary Table 1). Procedural blanks (Sterivex filters after filtration of 400 ml of autoclaved MilliQ water) were subjected to the same extraction procedures then treated as samples to detect potential contamination.

### ARISA

Automated Ribosomal Intergenic Spacer Analysis (ARISA) was carried out as a rapid method to compare microbial community structure across the water wells. ARISA-PCR was performed as previously described with primer sets 934f/71r and ITSf/ITSreub (Supplementary Table 3), targeting archaeal and bacterial 16S–23S rRNA gene intergenic regions, respectively (Vigneron et al., 2014b). One micro liter of each PCR reaction was analyzed on a DNA 7500 Chip using an Agilent 2100 Bioanalyzer according to the manufacturer's protocol (Agilent technology, Santa Clara, CA, USA). Data were recovered and normalized as previously detailed (Vigneron et al., 2014b), and Non-Metric Multidimensional Scaling was performed using PAST software (Hammer et al., 2001). No amplification was observed with the procedural blanks for DNA extraction.

### Quantitative PCR

The abundance of bacterial and archaeal 16S rRNA genes, methyl coenzyme M reductase genes (*mcrA*) of archaeal methanogens or anaerobic methanotrophs, and methane monooxygenase genes (*pmoA*) from aerobic methanotrophs was estimated using real-time quantitative PCR (qPCR) with the primer sets listed in Supplementary Table 3. Amplification reactions were performed in triplicate as previously described (Vigneron et al., 2016). Annealing temperatures for each assay are indicated in Supplementary Table 3. Standard curves from 10^2^ to 10^6^ copies of 16S rRNA genes and *mcrA* genes were prepared in triplicate with dilutions of genomic DNA from the strains listed in Supplementary Table 3. A standard curve for the *pmoA* gene was generated using dilutions of plasmids containing a *pmoA* gene cloned from an environmental sample. Three negative controls (autoclaved MilliQ water) were carried out with each experiment to evaluate potential contamination. The *R*^2^-values for standard curves obtained by real-time PCR were all >0.997 and PCR efficiencies are reported in Supplementary Table 3.

### Multigenic Miseq sequencing

To investigate the microbial communities involved in methane cycling, archaeal, and bacterial 16S rRNA, methyl coenzyme M reductase, and methane monooxygenase genes were analyzed using the primers described in Supplementary Table 3 with Miseq adaptors fused to the 5′ end of the primers. All PCR reactions were conducted in triplicate with the appropriate annealing temperature (Supplementary Table 3), as previously described (Vigneron et al., 2016). No amplification was observed in DNA extraction procedural blanks or PCR negative controls. Replicate amplicons were pooled and purified from agarose gels using a Qiagen MinElute Gel purification kit (Qiagen, Hilden, Germany). PCR products were indexed using a Nextera XT kit (Illumina Inc., San Diego, CA, USA) and diluted to equimolar concentration according to the manufacturer's recommendations. The DNA library was diluted to a concentration of 4 pM and sequenced using pair-end Illumina MiSeq sequencing. Sequencing was performed using an Illumina Miseq v3 kit (Illumina Inc.), as recommended by the manufacturer, to obtain 2 × 300 bp pair-end sequences.

After sequencing, datasets were split into reads from individual indexed amplicons *in silico* using Miseq Reporter™ software (Illumina Inc.). Reads were assembled into single pair-end sequences which were curated using QIIME version 1.9.1 (Caporaso et al., 2010). Sequences with low quality scores or flagged as chimeras were removed. Alignment and determination of the taxonomic affiliation of the reads were carried out using Silva release 119 (Pruesse et al., 2007), a publicly available *pmoA* database (Dumont et al., 2014), and an in-house generated *mcrA* sequence database containing more than 370 informative *mcrA* sequences (Supplementary Data). For *mcrA* database generation, taxonomic affiliations of uncultured *mcrA* sequences were determined using maximum-likelihood and neighbor-joining based phylogenetic trees as previously detailed (Cruaud et al., 2015). Statistical analyses (PCA and Bray-Curtis similarity clustering) of the sequences dataset were carried out using PAST software (Hammer et al., 2001). Raw sequences were deposited in the Genbank short read archive under PRJNA325306 BioProject (https://www.ncbi.nlm.nih.gov/bioproject/?term=325306).

## Results

### Geochemical characteristics of the water wells

**Figure 2:**
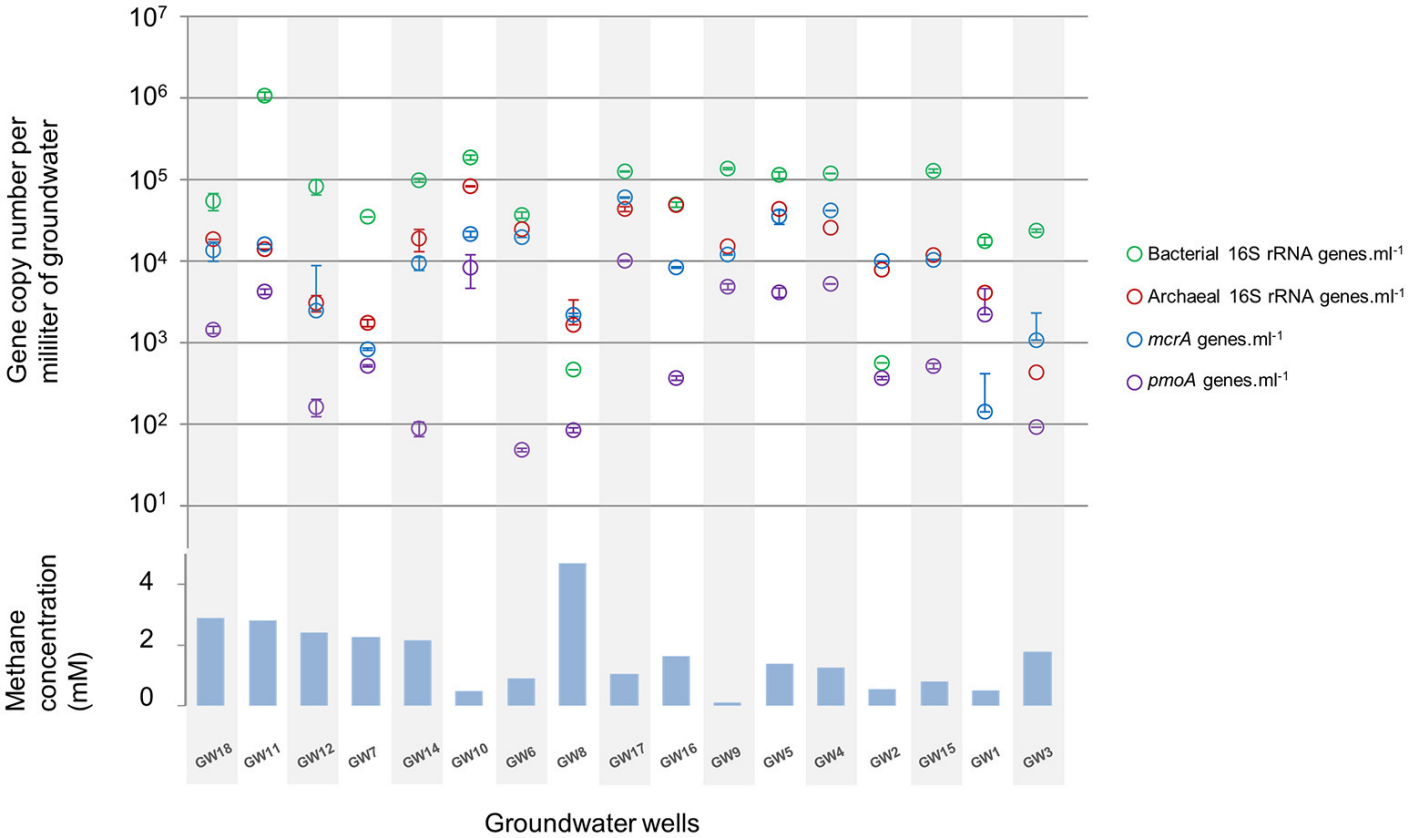
**Relative abundance of bacterial (green) and archaeal (red) rRNA gene, ***mcrA*** (blue) and ***pmoA*** (purple) genes per milliliter of water in groundwater samples**. The order of the wells corresponds to the Bay Curtis-based clustering of the samples according to their microbial community composition. Methane concentration (mM) in water samples at the time of the microbial sampling.

A total of 18 different water wells in the Pennsylvania region were sampled. Methane was detected in all samples with concentration ranging from 0.48 to 4.69 mM of CH_4_ in samples obtained at the time of sampling for microbial community analysis in 2015 (Supplementary Table 2, **Figure 2**). The carbon and deuterium isotopic signature of the methane in the samples obtained in 2015, ranged from −33.5 to −56.36‰ and −135 to −227‰, respectively. These isotopic values indicated a mixed thermogenic/biogenic origin for the methane (Figure 1A). The methane isotope data from 2015, also indicated that potentially, methane was being oxidized in the aquifers, leading to the relatively heavy isotopic signatures of the residual methane (Whiticar, 1999). Six of the water wells (GW1,2,9,15,17, and GW18) contained methane with a heavier isotopic signature (−33.5 to −37.88‰).

The geochemistry of water samples obtained over a 4 year period from 2011 to 2014 was also analyzed. These data provided the general context of the geochemical conditions in the aquifer system (Supplementary Figure 2). In samples taken between 2011 and 2014 methane was present in sub- to low-millimolar concentration (Supplementary Table 2). This was consistent with the levels of methane detected in 2015, in samples that were contemporaneous with the sampling for microbial community analysis. In addition to methane, in samples from 2011 to 2014, the hydrocarbon gases ethane (C_2_), ethane, and propane (C_3_) were detected in trace concentrations (<4.36 μM; Supplementary Table 2). The highest concentrations of non-methane gases were detected in wells GW1,2,9,15,17, and GW18. The presence of higher molecular weight hydrocarbon gases suggested that locally, larger amounts of thermogenic hydrocarbons were present in these samples (Figure 1B). The difference in methane concentration between sampling times and initial geochemical measurements as well as the observed fluctuations of methane and oxygen concentrations with time (Supplementary Figure 2) indicated considerable temporal variation in the geochemical environment. At the time of sampling for geochemical analysis, dissolved oxygen concentrations ranged from 0.04 to 0.33 mM whereas sulfate and nitrate were only detected in 5 and 3 of 18 wells, respectively (Supplementary Table 2).

### Microbial abundance and diversity in aquifers

**Figure 3:**
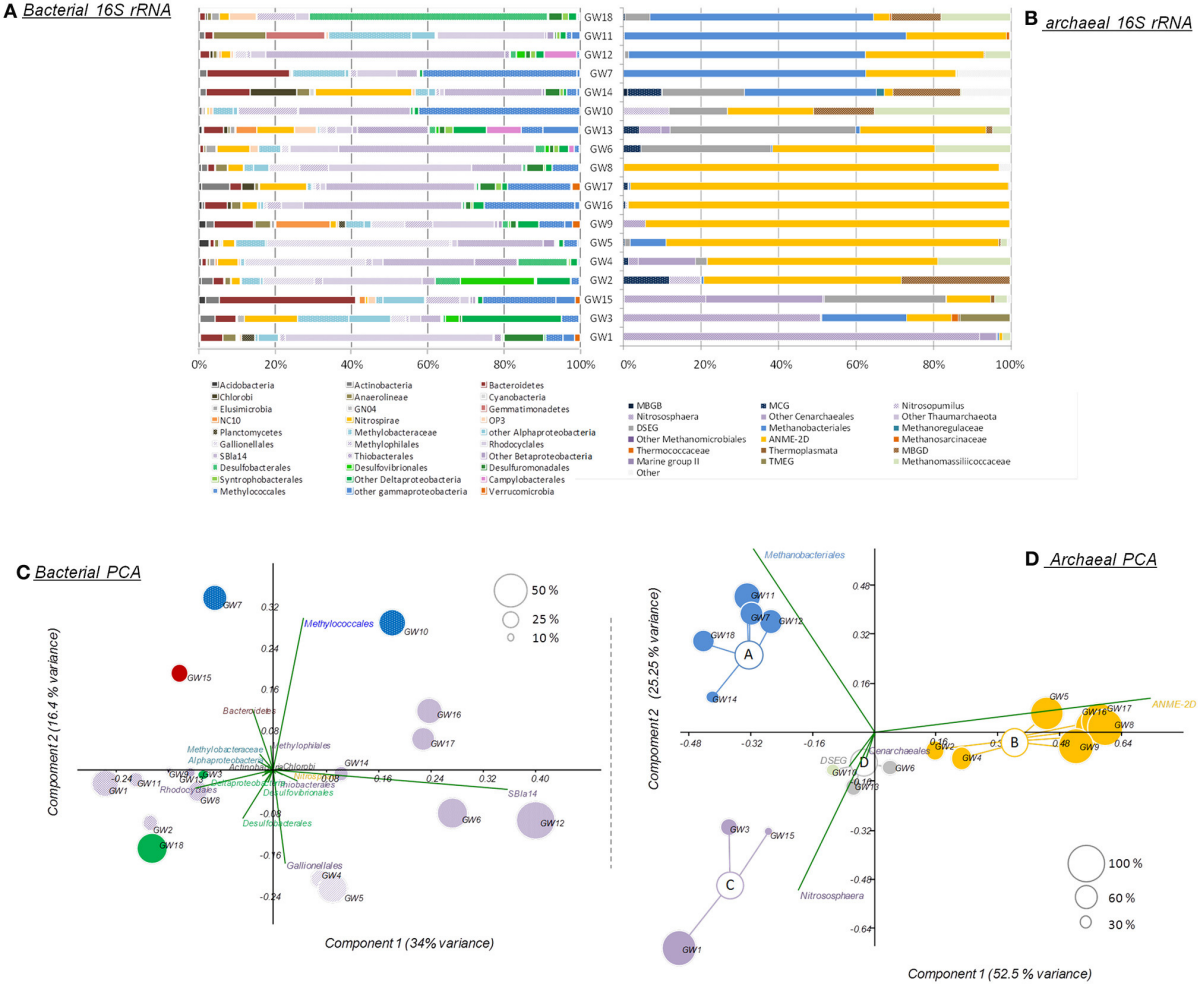
**(A)** Bacterial and **(B)** archaeal phylogenetic affiliations of 16S rRNA genes identified in the samples. Principal component analysis of **(C)** bacterial and **(D)** archaeal 16S rRNA gene sequencing datasets. Size and color of the dots reflect the proportions and the taxonomic affiliations of the predominant reads. MBGB/D, Marine Benthic Group B/D; MCG, Miscellaneous Crenarchaeotal Group; DSEG, Deep Sea Euryarchaeotal Group; ANME-2d, Anaerobic Methanotrophs-2d; TMEG, terrestrial Miscellaneous Euryarchaeotal Group. The order of the wells corresponds to the Bay Curtis-based clustering of the samples according to their microbial community composition.

The microbial abundance and community diversity in 18 different water wells containing methane and other hydrocarbon gases (Supplementary Table 2) from the Pennsylvania region were estimated by qPCR, 16S–23S intergenic spacer analysis, and 16S rRNA gene sequencing with an average of 9.05 ± 6.01 × 10^4^ reads per sample. Overall, bacterial and archaeal abundance were variable across the samples, ranging from 2.1 × 10^3^ to 1.08 × 10^6^ 16S rRNA gene copies per milliliter of water (Figure 2). Making the assumption that Bacteria and Archaea have the same average 16S rRNA gene copy number, bacteria represented 80 ± 14% of the microorganisms quantified in the ground water samples with an average of 1.31 × 10^5^ bacterial 16S rRNA gene copies.ml^−1^. However, in the samples with the lowest microbial abundance (<10^4^ 16S rRNA gene copy.ml^−1^, *n* = 3), Archaea represented more than 80% of the microbes with 3.75 × 10^3^ 16S rRNA gene copies.ml^−1^. Bacterial 16S rRNA gene analysis (Figures 3A,C) and ARISA (Supplementary Figure 4A) indicated a large variability in the bacterial community composition of the samples. Bacterial communities exhibited variable, complex profiles with high diversity (1-D_Simpson_ = 0.81 ± 0.11). The bacterial communities were generally dominated by members of the *Proteobacteria*. Bacteria involved in sulfur cycling (*Delta-proteobacteria*), nitrogen cycling (Nitrospirales, Methylomirabiliales), and iron cycling (Gallionellales). Organisms involved in organic matter degradation and fermentation (*Bacteroidetes*, Clostridiales, Anaerolineales) were detected in all samples but with different relative proportions (Figures 3A,C). Putative aerobic methanotrophs were detected in the water samples. Members of the *Methylococcaceae* (*Gammaproteobacteria*) were detected at considerable relative abundance in the water samples (9.2% on average, up to 41% of reads in libraries from sample GW10). Additionally, members of the Methylomirabiliales (NC10) (0.9% on average, up to 9.6% of reads in libraries from sample GW9) and *Methylocystaceae* (*Alphaproteobacteria*) (<1% in all samples) lineages were identified to a lesser extent in all samples. Potential methanol oxidizers belonging to the *Methylobacteraceae*, Methylophiliales, and related to *Methyloversatilis* (Rhodocyclales) lineages were also detected at substantial relative abundance in all samples (5–43% of the reads).

Archaeal 16S rRNA gene (Figures 3B,D) and ARISA (Supplementary Figure 4B) datasets also highlighted strong variability between wells however Bray-Curtis and Morisita Horn clustering highlighted four different archaeal community clusters (ANOSIM, *R* > 0.7, *P* < 0.05; Figure 3). Group A had a relatively low diversity (1-D_Simpson_ = 0.57) and was dominated by methanogenic lineages of the Methanobacteriales (*n* = 5, 58 ± 14% of the reads on average). Group B harbored a lower diversity (1-D_Simpson_ = 0.25), and was strongly dominated by sequences from an ANME-2d Methanosarcinales lineage (*n* = 7, 84 ± 19% of the reads), previously detected in rice paddy field samples, and in incubations showing evidence of denitrifying anaerobic methanotrophs (Raghoebarsing et al., 2006). Group C (1-D_Simpson_ = 0.52) was dominated by *Thaumarchaeota* most closely related to ammonia-oxidizing members of the phylum (*Nitrososphaera* and *Nitrosopumilus*) (*n* = 3, 53 ± 35% of the reads). Group D was more diverse (1-D_Simpson_ = 0.70) included members of the uncultured Deep Sea Euryarchaeotal Group (DSEG) (*n* = 3, 32 ± 16% of the reads) lineage (Figure 3). SIMPER analysis indicated that ANME-2d (31.79% contribution), Methanobacteriales (21.51% contribution), *Nitrososphaera* lineages (13.97% contribution), DSEG (10.7% contribution), and members of the *Methanomassiliicoccaceae* (6.59% contribution) together explained up to 85% of the difference observed between clusters of samples. Additionally, other uncultured lineages such as the Miscellaneous Crenarchaeotal Group (MCG), belonging to the *Bathyarchaeota* phylum and Marine Benthic Group D (also known as DHVEG-1) were detected in some samples (Figure 3B).

### Methane cycling archaea

**Figure 4:**
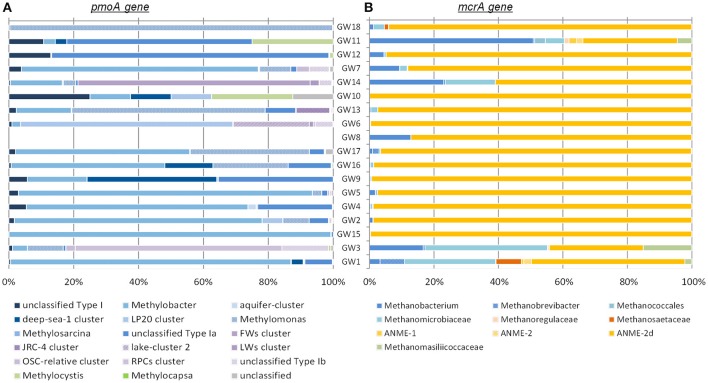
**Phylogenetic affiliations of (A)** *pmoA* and **(B)** *mcrA* genes detected in samples. The order of the wells corresponds to the Bay Curtis-based clustering of the samples according to their microbial community composition.

Diversity and abundance of methyl co-enzyme M reductase genes (*mcrA*), involved in both methane production and anaerobic methane oxidation in Archaea (Knittel and Boetius, 2009) were investigated by high throughput sequencing with an average of 5 ± 3.9 × 10^4^ reads per sample, and quantitative PCR. All water well samples contained detectable *mcrA* genes (Figure 4B). Quantification of *mcrA* gene abundance by qPCR highlighted that methanogens/ANME represented a large proportion of the Archaea in samples from groups A, B and D with more than 50% for A and, 80% for B and D, with up to 6 × 10^4^ *mcrA* copies per milliliter of water from sample GW5. Conversely in samples GW15 and GW1 from group C, methane cycle archaea represented 25 and 4% of the archaea respectively with as few as to 1.4 × 10^2^ *mcrA* copies per milliliter (Figure 2). A higher diversity of methanogens/ANME was detected in samples in groups A and C (1-D_Simpson_ = 0.38), where the recovered *mcrA* sequences were affiliated to ANME-2d, Methanobacteriales (*Methanobacterium* and *Methanobrevibacter*), and uncultured Methanomicrobiales and Methanomassiliicoccales lineages. By contrast, a very low diversity of methanogens/ANME was observed in group B and D samples (1-D_Simpson_ = 0.05) with 98% of the *mcrA* reads affiliated with ANME-2d related environmental sequences (Figure 4B).

### Methane cycling bacteria

Diversity and abundance of the gene encoding the alpha subunit of the methane mono-oxygenase (*pmoA*), involved in methane oxidation were investigated by high throughput sequencing with an average of 4.4 ± 2.9 × 10^3^ reads per sample, and quantitative PCR. Particulate methane monoxygenase alpha subunit genes were detected in all samples however for sample GW8, insufficient PCR product was obtained for sequencing (Figure 4A). Quantitative PCR targeting *pmoA* indicated that aerobic methanotrophs represented a small proportion (2.19 ± 2.37%) of the bacterial population, except in samples GW2 (65.31%), GW1 (12.55%), and GW10 (18%) with on average 2.5 × 10^3^ *pmoA* copies per milliliter of water (Figure 2). Sequences from both Type I (*Gammaproteobacteria*) and Type II (*Alphaproteobacteria*) methanotrophs were identified. Type I sequences were predominant and were mainly related to *Methylobacter* and *Methylomonas* as well as various environmental clusters (Deep-sea 1, LP20, Lake Cluster, or FW cluster) depending on the sample. Type II methanotroph sequences, related to *Methylocystis*, were also identified but these represented a minority of sequences. Even though 16S rRNA gene data indicated that bacteria related to NC10/*Methylomirabiliales* were present in some samples, no *pmoA* sequences from the NC10/*Methylomirabiliales* family were detected. This was due to six mismatches between NC10 *pmoA* sequence and the primer used (Luesken et al., 2011).

## Discussion

Water wells from the Pennsylvania region (Tioga County), exhibiting measurable methane concentrations (up to 4.69 mM) and traces of ethane and propane dissolved in water, were analyzed in detail to investigate methane cycling microorganisms and therefore evaluate the origin and fate of methane in this ecosystem. Although, all water wells represented the uppermost aquifer at their location, no correlation between microbial community composition and location of the water wells or their relative proximity to each other was observed (Supplementary Figure 1). This supports observations of localized recharge and discharge and that wells were either within separate aquifer flow system or that the hydrogeological setting was highly complex. Significant fluctuations in oxygen and methane concentrations with time, and changes from aerobic to methanogenic conditions and *vice versa* were detected in three groundwater wells monitored over a long period (2011–2014, Supplementary Figure 2). Similar fluctuations are frequent in the region and were previously observed (Wilson, 2014). This confirms that environmental conditions in the aquifers are extremely dynamic (Datry et al., 2004; Griebler and Lueders, 2009). This temporal variability is probably due to recharge and discharge events, and is likely to affect the distribution of individual redox zones generally observed in groundwater habitats (Datry et al., 2004; Haack et al., 2004; Griebler and Lueders, 2009) and the microbial communities in shallow aquifers (Vroblesky and Chapelle, 1994). One can therefore postulate that different water table levels within the aquifers due to precipitation for example, could lead to the sampling of different redox zones (oxic or anoxic) and hence detection of microbial communities with different composition from the same well at different times. A consequence of the temporal dynamics observed is that no direct correlation can be made between microbial community composition and geochemical conditions measured at a different time. Therefore, we have used geochemical data from 2011 to 2014 to provide broad context for the groundwater environment under study and microbial community data is only discussed in the context of methane and isotope data which were obtained contemporaneously. The variability in groundwater geochemistry is consistent with the observed variability in microbial abundance and community composition observed by qPCR and multigenic sequencing of different water samples.

Periodic methanogenic conditions were observed between 2011 and 2014. Moreover, at the time of sampling for microbial community analysis, the systematic detection of methane and the carbon and hydrogen isotope measurements made, indicated that methane cycling is a major microbial process in the groundwater analyzed in this study. The occurrence of a range of methanogens and a dominant population of ANME, (detected by archaeal 16S rRNA and *mcrA* gene analysis) as well as aerobic methanotrophs and methylotrophs (detected by 16S rRNA and *pmoA* gene surveys) in some of the wells supports the inference that there is capacity for both biological methane generation and consumption.

### Methane origin in groundwater

Methane is a common trace constituent of groundwater (Barker and Fritz, 1981; Molofsky et al., 2011). Although, it has been detected in water wells of the Pennsylvania region for centuries (Molofsky et al., 2011, 2016), the origin of methane in aquifers and groundwater has remained misunderstood and it is likely that methane in the aquifers originates from a range of sources (Breen et al., 2007; Osborn et al., 2011; Wilson, 2014). Methane carbon and hydrogen isotopic ratios are frequently used to determine the possible source of methane in environmental samples (Whiticar, 1999). However, microbial activities can strongly alter the isotopic composition of methane (Barker and Fritz, 1981). Indeed, microbial oxidation of methane by both aerobic and anaerobic methanotrophs leads to the depletion of the lighter isotopes of methane and therefore to the enrichment of residual isotopically heavier methane, which can be thereafter misinterpreted as thermogenic gas (Barker and Fritz, 1981; Whiticar, 1999; Yoshinaga et al., 2014). Therefore, a careful analysis of the data as well as complementary approaches are required for a correct interpretation of methane sources. The isotopic signatures of the methane measured in the water samples seemed to indicate a dominant contribution from thermogenic methane (δ^13^C > −55‰), sourced from deeper sediments as previously suggested (Breen et al., 2007; Figure 1A). The detection of ethane and propane in wells GW1,2,9,15,17, and GW18 (Figure 1B) is consistent with this interpretation. However, the absence of higher molecular weight gaseous alkanes in the majority of the water samples (*n* = 11) did not support this hypothesis [C_1_/(C_2_ + C_3_) > 1,000; Figure 1B]. The heavy isotopic signature of methane, sampled at the same time as samples for microbial community analysis, coupled with the absence of C_2_ and C_3_ gases indicated that biogenic methane has likely undergone oxidation in most of the aquifers sampled (Humez et al., 2016). The isotope signature of methane therefore, probably has been influenced by the microbial activity and the proportion of biogenic methane may be underestimated owing to the oxidation of methane (Barker and Fritz, 1981). Methanogenic lineages affiliated to Methanobacteriales, Methanomicrobiales, and Methanomassiliicoccales were detected by 16S rRNA and *mcrA* gene sequencing, these were similar to methanogens identified in other hydrocarbon-rich groundwater (Kleikemper et al., 2005; Kotelnikova and Pedersen, 2006; Griebler and Lueders, 2009). The presence of these archaea highlights the metabolic potential for methane production in the aquifers sampled. Furthermore, a significant correlation was observed between methane concentration at the time of sampling for microbial community analysis and the relative representation of Methanobacteriales sequences in amplicons libraries (Pearson correlation *r* = 0.49, *P* = 0.05). This could indicate that the highest methane concentrations quantified in aquifers might be contributed to by the activity of methanogens rather than purely due to seepage of thermogenic methane from deeper reservoirs. Members of the Methanobacteriales are generally hydrogenotrophic, using H_2_ released from the fermentative metabolism of syntrophic partners, to reduce CO_2_ to CH_4_ (Bonin and Boone, 2006). Potential fermenters and syntrophic partners for methanogens, e.g., *Syntrophobacter* and *Desulfovibrio* were also detected in the samples by 16S rRNA gene sequencing. Thus, the methanogens present might grow syntrophically with fermentative bacteria by the degradation of organic carbon or hydrocarbons present in the aquifer. This is also consistent with the predominance of hydrogenotrophic methanogens in organic carbon poor aquifers (Kotelnikova, 2002). Detection of these methanogenic lineages, coupled with methane isotope data and the absence of higher alkanes indicate that for the majority of the wells a least part of the methane detected was produced microbially. By contrast, methane from water wells GW1,2,9,15,17, and GW18 are more likely to be from thermogenic origin.

### Methanotrophs and methane oxidation

Although, the concentration of higher alkanes may have changed between the time of the historical geochemical survey (2011–2014) and the microbiological investigation (2015), the heavy isotope signature of methane at the time of the microbial sampling does suggest that methane oxidation potentially occurs in groundwater, as previously inferred based on radiotracer studies (Hansen, 1998) and natural-gradient tracer tests (Smith et al., 1991). Both aerobic and anaerobic methanotrophs were identified consistently in water wells. Methanotrophs represented a sizeable proportion of the microbial community with up to 84% of the archaeal reads and 41% of the bacterial 16S rRNA gene sequences being assigned to ANME and aerobic methanotrophs, respectively. Some discrepancies between 16S rRNA gene and functional gene surveys were observed (e.g., a lower representation of methanogens in *mcrA* gene libraries). This may be due to difference in gene copy number or specificity of the different primers used to generate amplicons libraries and for qPCR. Overall, the relative abundance of *pmoA* and *mcrA* genes indicated that anaerobic methane oxidation was dominant in the water wells at the time of sampling, except in sample GW1 where *pmoA* genes were more abundant than *mcrA* genes. Consistent with this, the archaeal community of GW1 was strongly dominated by aerobic lineages from the *Thaumarchaeota* and the proportion of strictly anaerobic microbes such as methanogens and anaerobic methanotrophs was limited. These results suggest that well GW1 was oxic at the time of the sampling for microbial community analysis and that under oxic conditions aerobic methanotrophs can outcompete and replace ANME communities as the dominant methane oxidizers as has been previously observed in other aquifers (Erwin et al., 2005).

Both 16S rRNA and *mcrA* gene libraries indicated that anaerobic archaeal methanotrophs were affiliated to the ANME-2d lineage. These archaea, closely affiliated to other anaerobic methanotrophs (ANME-2a/b and c) within the Methanosarcinales lineage have been previously detected in cold seep sediments (GoM Arc-I in Lloyd et al., 2010; Vigneron et al., 2013), rice paddy field soil and activated sludge incubated under denitrifying conditions (Raghoebarsing et al., 2006). ANME-2d have also been observed in freshwater sediments (AOM Associated Archaea—AAA group in Schubert et al., 2011), in laboratory incubations (Timmers et al., 2016), and in other methane-rich aquifers (Flynn et al., 2013). Specific enrichment cultures with methane and nitrate demonstrated that ANME-2d couple anaerobic oxidation of methane to nitrate reduction with a methane consumption rate of up to 1.1 mmol of methane per day (Haroon et al., 2013). Furthermore, ANME-2d were also enriched in co-culture with NC10/*Methanomirabilis*-like bacteria (Raghoebarsing et al., 2006; Haroon et al., 2013), which were also detected in a number of water wells in this study by 16S rRNA gene sequencing (Figure 3A). In enrichments of ANME-2d and NC10/*Methanomirabilis*-like bacteria, ANME-2d oxidized methane by reducing nitrate to nitrite while NC10 bacteria oxidized methane by reducing the nitrite produced by ANME-2d population (Raghoebarsing et al., 2006; Haroon et al., 2013). This result suggests that anaerobic methane oxidation is probably coupled to nitrate and nitrite reduction in groundwater ecosystem.

Bacterial 16S rRNA and *pmoA* gene sequencing also identified aerobic methanotrophs, which have been reported in other aquifers (Newby et al., 2004; Erwin et al., 2005). Although, the diversity of aerobic methanotroph might be overestimated by *pmoA* gene surveys due to divergent gene copies in a single taxonomic group, aerobic methanotrophs detected in groundwater samples were mainly affiliated with Type I methanotrophic lineages from the Methyloccocales (Bowman, 2006). However, some members of the Methyloccocales such as *Methylomonas*, identified in the water wells by *pmoA* gene sequencing, can couple methane oxidation to nitrate reduction (Kits et al., 2015), suggesting that in groundwater the aerobic part of the methane cycle can be also associated with the nitrogen cycle as previously observed in batch incubations (Eisentraeger et al., 2001). Particulate methane monooxygenase alpha subunit sequences affiliated to environmental clusters were also detected in the water wells. Sequences belonging to these environmental clusters were previously detected in aquatic environments, such as aquifers (LP20, aquifer-cluster), freshwater lakes (LWs, FWs, lake-cluster), and deep sea samples (deep-sea cluster), suggesting the occurrence of novel freshwater adapted lineages of aerobic methanotrophs in aquifers. Additionally, other methylotrophic lineages (*Methylobacteraceae*, Methylophiliales, and *Methyloversatilis*) were identified in 16S rRNA gene libraries. Cultivated representatives of these bacterial lineages grow on methanol as a carbon source. Methanol is an intermediate of aerobic methane oxidation, therefore these methylotrophs may use by-products of methanotrophs metabolism and complete the oxidation of some methane in groundwater. Furthermore, members of the Methylophiliales, and organisms related to *Methyloversatilis* were found to couple methanol oxidation to denitrification (Baytshtok et al., 2009; Kalyuhznaya et al., 2009), supporting the potential link between the methane and nitrogen cycles in these groundwater ecosystems.

### A comparison of freshwater and marine methane seeps

Knowledge of methane and hydrocarbon seeps is largely based on studies of marine ecosystems and their counterpart in groundwater environments remain poorly explored. In marine sediments, the methane cycle is generally linked to the sulfur cycle. Anaerobic methanotrophs (ANME-1, ANME-2a/b/c, and ANME-3) form microbial consortia with sulfate-reducing *Deltaproteobacteria* (SEEP SRB1, SEEP SRB2, and *Desulfobulbus* related lineages; Orphan et al., 2002; Knittel et al., 2005; Niemann et al., 2006) where methane oxidation is coupled to sulfate reduction or S^0^ disproportionation (Milucka et al., 2012). Additionally, aerobic methane oxidation has a minor role compared with anaerobic oxidation in these marine seep environments (1–3% of total methane oxidation; Niemann et al., 2006). By contrast, in the aquifer samples analyzed in this study, methane oxidation appears to be linked with the nitrogen cycle. Indeed, ANME-2d but also NC10, *Methylomonas* and other methylotrophic lineages (Methylophiliales, *Methyloversatilis*), detected in the aquifer samples, are known to couple methane and methanol oxidation to nitrate and nitrite reduction or denitrification. Nitrate concentrations were below the detection limit for the majority of the water wells analyzed in initial geochemical investigations between 2011 and 2014, suggesting that turnover of nitrate in the aquifer is considerable. However, nitrifying bacteria (*Nitrospirae*) and archaea (*Nitrososphaera, Nitrosopumilus*, and other *Thaumarchaeota*) were identified in the aquifers by 16S rRNA gene sequencing, suggesting that in the oxic regions of the aquifers or during periods when oxic conditions prevail, nitrate and nitrite can be produced in the groundwater by microbial ammonia oxidation. Ammonia might have different origins in groundwater. Surface contamination by agricultural fertilizers, landfill leachate, and wastewater disposal are frequently the main sources of ammonia in groundwater, however subsurface organic rich zones such as shales might also provide significant amount of ammonia in aquifer environments (Buss et al., 2004).

Taken together, these results highlighted that, in addition to migration from subsurface reservoirs, methane in aquifers of the Pennsylvania region can also be produced locally by methanogenic archaea. Moreover, and consistent with methane isotope data, there was evidence of considerable potential for microbial methane oxidation with the occurrence of abundant anaerobic methanotrophs affiliated with the ANME-2d lineage, bacteria from the NC10 phylum, Type I aerobic methanotrophs and various methylotrophs, indicating that the fate of methane in groundwater ecosystems is closely coupled to the nitrogen cycle. These results also demonstrated that the groundwater microbial community had the genetic potential for bio-attenuation of methane. However, in addition to the isotopic data reported here, *in situ* activity measurements of methane oxidation will be required to determine the kinetics of methane oxidation and thus the feasibility of promoting methane oxidation in methane-containing aquifers.

## Author contributions

AV, EA, KH, IH, and NT designed, performed, analyzed, and interpreted the experiments. AV, EA, IH, and NT wrote the manuscript. AB, IR, and RH contributed to data interpretation and revised the manuscript.

## Funding

This work was funded by Shell International Exploration and Production Inc.

### Conflict of interest statement

The authors declare that the research was conducted in the absence of any commercial or financial relationships that could be construed as a potential conflict of interest.

## Supplementary material

Supplementary Table 1 **DNA isolation procedure, microbial abundance and diversity indexes**.

Supplementary Table 2 **Geochemical characteristics of the ground water samples**.

Supplementary Table 3 **PCR primers used for PCR, ARISA and real-time quantitative PCR**.

Supplementary Figure 1 **Schematic map of the sampled wells (Altitude of the well/well depth in meters)**.

Supplementary Figure 2 **Time series of dissolved oxygen (green) and methane (blue) proportion in water wells GW2, GW1, and GW9 between 2011 and 2014**. Data for other wells are not available.

Supplementary Figure 3 **Diagram representing the sampling history of the water wells**.

Supplementary Figure 4 **Non-Metric Multidimensional Scaling of (A)** bacterial and **(B)** archaeal ARISA dataset.
