# Supplementary material for: Microbial and Isotopic Evidence for Methane Cycling in Hydrocarbon-Containing Groundwater from the Pennsylvania Region
Source: Front Microbiol. 2017 Apr 5;8:593. doi: 10.3389/fmicb.2017.00593 (PMC5380731; doi:10.3389/fmicb.2017.00593)
Supplement: Supplementary Table 1 — DNA isolation procedure, microbial abundance and diversity indexes. [file Table1.docx]

| Wells  ID | DNA Extraction procedure | Microbial abundance  (16S rRNA Copy/ml) | 1-D Simpson (Bacteria) | Shannon (Bacteria) | Chao-1 (Bacteria) | 1-D Simpson (Archaea) | Shannon (Archaea) | Chao-1 (Archaea) |
| --- | --- | --- | --- | --- | --- | --- | --- | --- |
| GW1 | PowerWater DNA Isolation Kit | 2.17E+04 | 0.804 | 2.629 | 190 | 0.154 | 0.3887 | 11 |
| GW2 | PowerWater DNA Isolation Kit | 8.40E+03 | 0.8724 | 2.587 | 104 | 0.6409 | 1.217 | 9 |
| GW3 | PowerWater DNA Isolation Kit | 2.43E+04 | 0.8917 | 2.698 | 37 | 0.6642 | 1.33 | 11 |
| GW4 | PowerWater DNA Isolation Kit | 1.40E+05 | 0.8377 | 2.42 | 195 | 0.5881 | 1.177 | 9 |
| GW5 | PowerWater DNA Isolation Kit | 1.69E+05 | 0.7449 | 2.177 | 140 | 0.2549 | 0.5744 | 9 |
| GW6 | PowerWater DNA Isolation Kit | 3.06E+04 | 0.751 | 2.323 | 83 | 0.6718 | 1.219 | 8 |
| GW7 | PowerWater DNA Isolation Kit | 1.08E+06 | 0.7956 | 2.145 | 85 | 0.5368 | 0.9433 | 6 |
| GW8 | PowerWater DNA Isolation Kit | 1.44E+05 | 0.8529 | 2.488 | 87 | 0.06162 | 0.1442 | 4 |
| GW9 | PowerWater DNA Isolation Kit | 9.82E+04 | 0.9571 | 3.726 | 156 | 0.1144 | 0.2431 | 4 |
| GW10 | PowerWater DNA Isolation Kit | 2.12E+03 | 0.7207 | 1.717 | 83 | 0.7672 | 1.548 | 8 |
| GW11 | PowerWater DNA Isolation Kit | 2.10E+03 | 0.8618 | 2.414 | 61 | 0.4041 | 0.6569 | 6 |
| GW12 | PowerLyzer PowerSoil DNA Isolation Kit | 8.51E+04 | 0.6403 | 2.027 | 180 | 0.5293 | 0.9353 | 11 |
| GW13 | PowerLyzer PowerSoil DNA Isolation Kit | NA | 0.9462 | 3.637 | 161 | 0.6573 | 1.368 | 10 |
| GW14 | PowerLyzer PowerSoil DNA Isolation Kit | 7.30E+04 | 0.865 | 2.725 | 175 | 0.7845 | 1.723 | 10 |
| GW15 | PowerLyzer PowerSoil DNA Isolation Kit | 1.35E+05 | 0.9231 | 3.239 | 239 | 0.7529 | 1.56 | 12 |
| GW16 | PowerLyzer PowerSoil DNA Isolation Kit | 1.52E+05 | 0.79 | 2.409 | 157 | 0.03408 | 0.1179 | 11 |
| GW17 | PowerLyzer PowerSoil DNA Isolation Kit | 1.57E+05 | 0.8223 | 2.545 | 144 | 0.04889 | 0.156 | 11 |
| GW18 | PowerLyzer PowerSoil DNA Isolation Kit | 1.17E+05 | 0.7266 | 2.016 | 126 | 0.613 | 1.255 | 10 |

**Supplementary Table 1**: DNA isolation procedure, microbial abundance and diversity indexes.
