# Supplementary material for: Microbial and Isotopic Evidence for Methane Cycling in Hydrocarbon-Containing Groundwater from the Pennsylvania Region
Source: Front Microbiol. 2017 Apr 5;8:593. doi: 10.3389/fmicb.2017.00593 (PMC5380731; doi:10.3389/fmicb.2017.00593)
Supplement: Supplementary Table 2 — Geochemical characteristics of the ground water samples. [file Table2.DOCX]

| Wells  ID | pH |  | T.O.C. mg/L | Methane^#^ (mM) | δDC1^#^  (‰) | δ13C1^#^ (‰) | Sulfate (mM) | Nitrate (mM) | O_2_  (mM) | CO_2_*  (mM) | N_2_*  (mM) | CO*  (mM) | C_1_*  (mM) | C_2_*  (µM) | C_2_H_4_*  (µM) | C_3_*  (µM) |
| --- | --- | --- | --- | --- | --- | --- | --- | --- | --- | --- | --- | --- | --- | --- | --- | --- |
| GW1 | 7.51 |  | 0 | 1.79 | -179.8 | -37.51 | <0.05 | <0.02 | 0.08* | 1.56* | 0.07* | 0.00* | 1.93* | 4.36* | 0.00* | 0.01* |
| GW2 | 7.98 |  | 0 | 0.56 | -173.9 | -37.88 | 0.21 | <0.02 | 0.05* | 3.39* | 0.38* | 0.00* | 0.87* | 0.36* | 0.00* | 0.00* |
| GW3 | 8.04 |  | 0.589 | 0.51 | -206.6 | -49.18 | <0.05 | <0.02 | 0.26^+^ | 1.91^+^ | 0.40^+^ | 0.00^+^ | 0.43^+^ | 0.01^+^ | 0.00^+^ | 0.00^+^ |
| GW4 | 7.69 |  | 1.67 | 1.26 | -203.4 | -46.59 | 0.08 | <0.02 | 0.10^α^ | 12.34 ^α^ | 0.29 ^α^ | 0.00 ^α^ | 1.02 ^α^ | 0.01 ^α^ | 0.00 ^α^ | 0.00 ^α^ |
| GW5 | 7.75 |  | 0.69 | 1.38 | -212.5 | -46.6 | <0.05 | 0.03 | 0.05 ^α^ | 3.32 ^α^ | 0.31 ^α^ | 0.00 ^α^ | 1.12 ^α^ | 0.02 ^α^ | 0.00 ^α^ | 0.00 ^α^ |
| GW6 | 7.80 |  | 0.675 | 0.90 | -224.2 | -49.32 | <0.05 | <0.02 | NA | NA | NA | NA | NA | NA | NA | NA |
| GW7 | 7.46 |  | 0.64 | 2.26 | -208.9 | -41.61 | <0.05 | <0.02 | 0.25 ^α^ | 1.50 ^α^ | 0.29 ^α^ | 0.00 ^α^ | 0.86 ^α^ | 0.03 ^α^ | 0.00 ^α^ | 0.00 ^α^ |
| GW8 | 7.76 |  | 0.623 | 4.69 | -220.7 | -46.69 | <0.05 | <0.02 | 0.30 ^α^ | 0.12 ^α^ | 0.50 ^α^ | 0.00 ^α^ | 0.04 ^α^ | 0.00 ^α^ | 0.00 ^α^ | 0.00 ^α^ |
| GW9 | 6.38 |  | 0 | 0.11 | -135 | -33.5 | 0.06 | <0.02 | 0.24^γ^ | 3.81 ^γ^ | 0.48 ^γ^ | 0.00 ^γ^ | 0.17 ^γ^ | 0.26 ^γ^ | 0.00 ^γ^ | 0.00 ^γ^ |
| GW10 | 7.86 |  | 0.695 | 0.48 | -227.6 | -56.36 | <0.05 | <0.02 | 0.31 ^α^ | 1.96 ^α^ | 0.41 ^α^ | 0.00 ^α^ | 0.31 ^α^ | 0.00 ^α^ | 0.00 ^α^ | 0.00 ^α^ |
| GW11 | 7.77 |  | 0.668 | 2.81 | -209.5 | -43.34 | <0.05 | <0.02 | 0.20 ^α^ | 0.92^α^ | 0.37 ^α^ | 0.00 ^α^ | 0.69 ^α^ | 0.02 ^α^ | 0.00 ^α^ | 0.00^α^ |
| GW12 | 7.72 |  | 0.72 | 2.42 | -207.9 | -43.53 | <0.05 | <0.02 | 0.04 ^α^ | 3.08 ^α^ | 0.25 ^α^ | 0.00 ^α^ | 1.36 ^α^ | 0.03 ^α^ | 0.00 ^α^ | 0.00 ^α^ |
| GW13 | 7.58 |  | 0.624 | 0.75 | -225.5 | -51.9 | <0.05 | <0.02 | 0.33 ^α^ | 2.44^α^ | 0.41 ^α^ | 0.00 ^α^ | 0.27 ^α^ | 0.01^α^ | 0.00 ^α^ | 0.00 ^α^ |
| GW14 | 7.73 |  | 0 | 2.16 | -212.5 | -43.97 | <0.05 | 0.02 | 0.22^β^ | 1.72 ^β^ | 0.21 ^β^ | 0.00 ^β^ | 1.22 ^β^ | 0.03 ^β^ | 0.00 ^β^ | 0.00 ^β^ |
| GW15 | 7.28 |  | 0 | 0.80 | -162.5 | -34.47 | 0.17 | <0.02 | 0.06 ^γ^ | 2.71 ^γ^ | 0.10 ^γ^ | 0.00 ^γ^ | 1.83 ^γ^ | 3.95 ^γ^ | 0.00 ^γ^ | 0.08 ^γ^ |
| GW16 | 7.85 |  | 0.836 | 1.63 | -211 | -44.6 | <0.05 | <0.02 | 0.12 ^α^ | 1.72^α^ | 0.21^α^ | 0.00 ^α^ | 1.37 ^α^ | 0.03 ^α^ | 0.00 ^α^ | 0.00 ^α^ |
| GW17 | 7.81 |  | 0 | 1.05 | -168.8 | -41.43 | <0.05 | 0.02 | 0.27^ε^ | 1.74 ^ε^ | 0.30 ^ε^ | 0.00 ^ε^ | 0.77 ^ε^ | 1.79 ^ε^ | 0.00 ^ε^ | 0.03 ^ε^ |
| GW18 | 7.55 |  | 0 | 2.89 | -160.4 | -34.82 | 0.20 | <0.02 | 0.20 ^β^ | 0.22 ^β^ | 0.34 ^β^ | 0.00 ^β^ | 0.76 ^β^ | 1.76 ^β^ | 0.00 ^β^ | 0.07 ^β^ |

**Supplementary Table 2**: Geochemical characteristics of the ground water samples.

T.O.C. : Total Organic Carbon, NA : not available, ^#^: Measured at the microbial sampling time, δDC1: Deuterium isotopic signature of the methane, δ13C1: Carbon isotopic signature of the methane, *: measured on Sept. 2014, ^+^: measured on Feb. 2014, ^α^: measured on Nov. 2013, ^β^ : measured on Aug. 2011, ^γ^: measured on Mar. 2014, ^ε^: measured on Sept. 2011
