# Supplementary material for: Microbial and Isotopic Evidence for Methane Cycling in Hydrocarbon-Containing Groundwater from the Pennsylvania Region
Source: Front Microbiol. 2017 Apr 5;8:593. doi: 10.3389/fmicb.2017.00593 (PMC5380731; doi:10.3389/fmicb.2017.00593)
Supplement: Supplementary Table 3 — PCR primers used for PCR, ARISA and real-time quantitative PCR. [file Table3.docx]

**Supplementary Table 3.** PCR primers used for PCR, ARISA and real-time quantitative PCR

| Name | **Function** | **Target group** | **Sequence (5' - 3')** | **Amplicon size (bp)** | **Annealing Temp. (°C)** | **Strain for Standard** | **Efficiency (%)** | **Ref.** |
| --- | --- | --- | --- | --- | --- | --- | --- | --- |
| 934f  71r | ARISA | *Archaea* | AGG-AAT-TGG-CGG-GGG-AGC-A  TCG-GYG-CCG-AGC-CGA-GCC-ATC-C | Variable | 55 | - | - | (Casamayor et al., 2002) |
| ITSf  ITSreub | ARISA | *Bacteria* | GTC-GTA-ACA-AGG-TAG-CCG-TA  GCC-AAG-GCA-TCC-ACC | Variable | 55 | *-* | - | (Cardinale et al., 2004) |
| BACT1369F  BACT1492R | Q-PCR | *Bacteria* | CGG-TGA-ATA-CGT-TCY-CGG  GGW-TAC-CTT-GTT-ACG-ACT-T | 142 | 60 | *Desulfobulbus*  *DSM 2032* | 99 | ([Suzuki et al., 2000](#_ENREF_5)) |
| ARC787F  ARC1059R | Q-PCR | *Archaea* | ATT-AGA-TAC-CCS-BGT-AGT-CC  GCC-ATG-CAC-CWC-CTC-T | 273 | 60 | *Methanococcoides DSM6242* | 95 | ([Yu et al., 2005](#_ENREF_9)) |
| S-D-Bact-0516-a-S-18  S-D-Bact-0907-a-A-20 | Sequencing | Bacteria | TGS-CSG-CSG-CCG-CCG-TAA  CCG-TCA-ATT-CMT-TTG-AGT-TT | 420 | 58 | - | - | (Klinworth et al.,2012 |
| S-D-Arch-0008-b-S-18  S-D-Arch-0519-a-A-19 | Sequencing | *Archaea* | TCY-GGT-TGA-TCC-TGS-CGG  GGT-DTT-ACC-GCG-GCK-GCT-G | 530 | 58 | - | - | (Klinworth et al.,2012) |
| MLf  MLr | Sequencing  Q-PCR | McrA | GGT-GGT-GTM-GGA-TTC-ACA-CAR-TAY-GCW-ACA-GC  TTC-ATT-GCR-TAG-TTW-GGR-TAG-TT | 550 | 55 | *Methanococcoides DSM6242* | 73 | (Luton et al. 2002) |
| A189f  MB661r | Sequencing  Q-PCR | pmoA | GGN-GAC-TGG-GAC-TTC-TGG  CCG-GMG-CAA-CGT-CYT-TAC-C | 508 | 55 | Environmental clone | 95 | (McDonald et al. 2008) |
| Adaptor F  Adaptor R | Sequencing | - | TCGTCGGCAGCGTCAGATGTGTATAAGAGACAG  GTCTCGTGGGCTCGGAGATGTGTATAAGAGACAG | - | - |  | - | - |
