# Supplementary figures and images for: Microbial and Isotopic Evidence for Methane Cycling in Hydrocarbon-Containing Groundwater from the Pennsylvania Region
Source: Front Microbiol. 2017 Apr 5;8:593. doi: 10.3389/fmicb.2017.00593 (PMC5380731; doi:10.3389/fmicb.2017.00593)

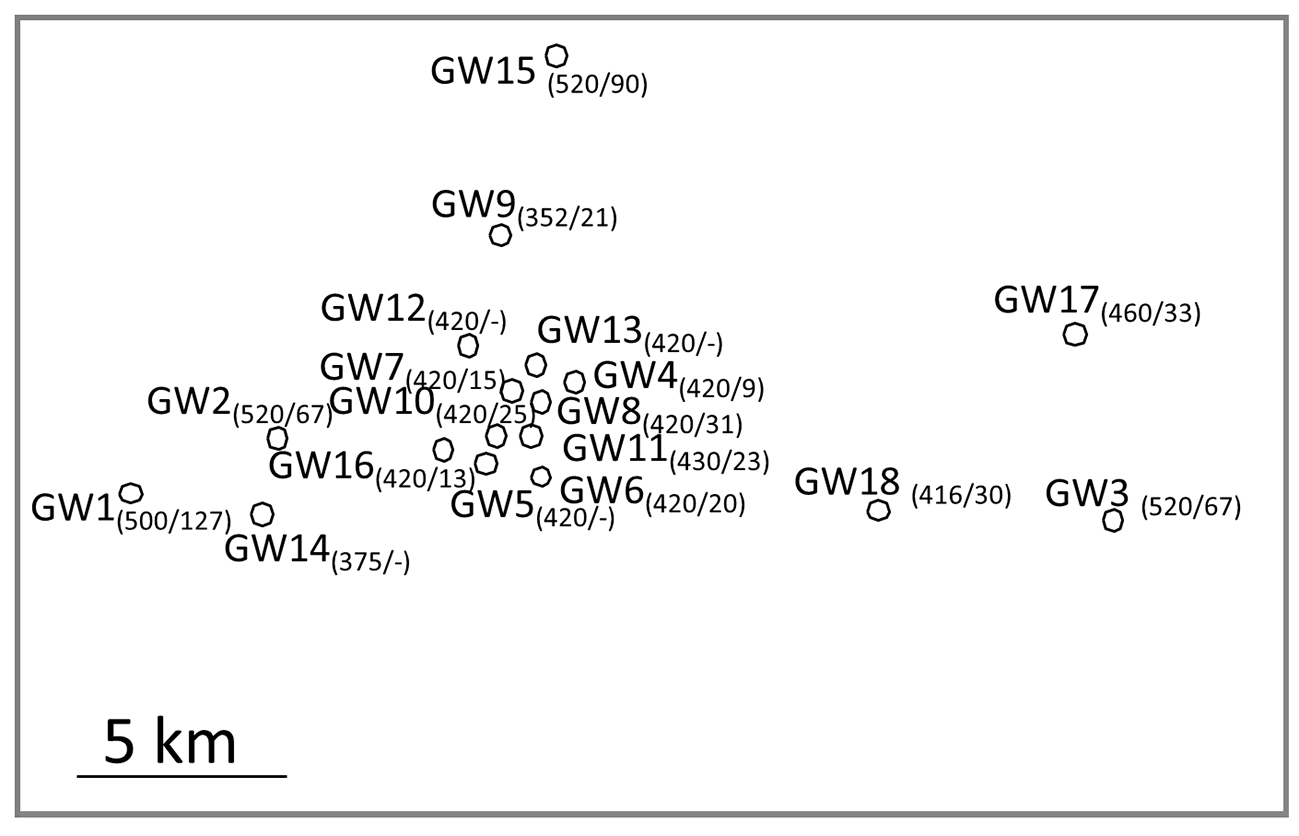

Supplement: Supplementary Figure 1 — Schematic map of the sampled wells (Altitude of the well/well depth in meters). [file Image1.TIF]

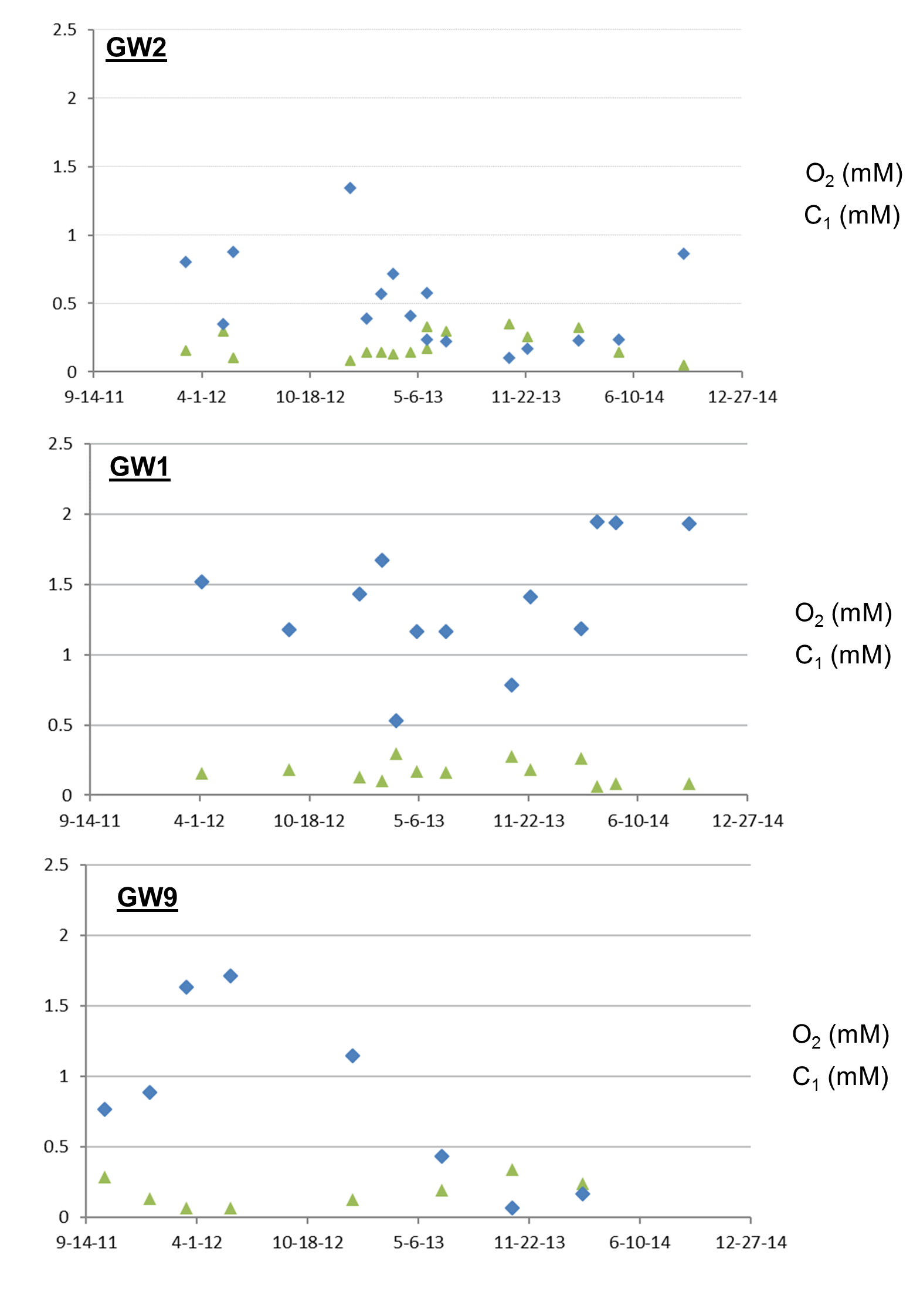

Supplement: Supplementary Figure 2 — Time series of dissolved oxygen (green) and methane (blue) proportion in water wells GW2, GW1, and GW9 between 2011 and 2014. Data for other wells are not available. [file Image2.TIF]

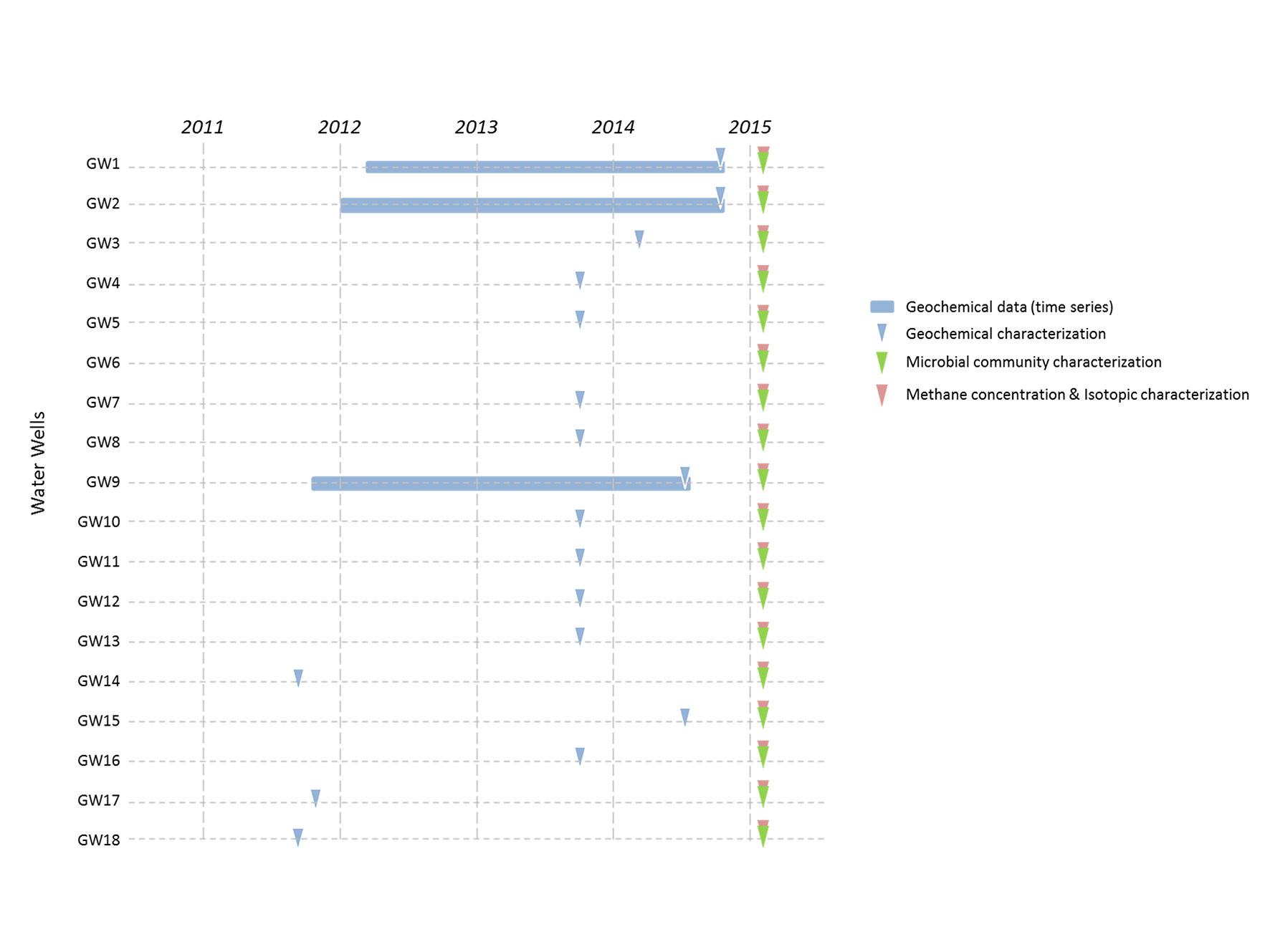

Supplement: Supplementary Figure 3 — Diagram representing the sampling history of the water wells. [file Image3.TIF]

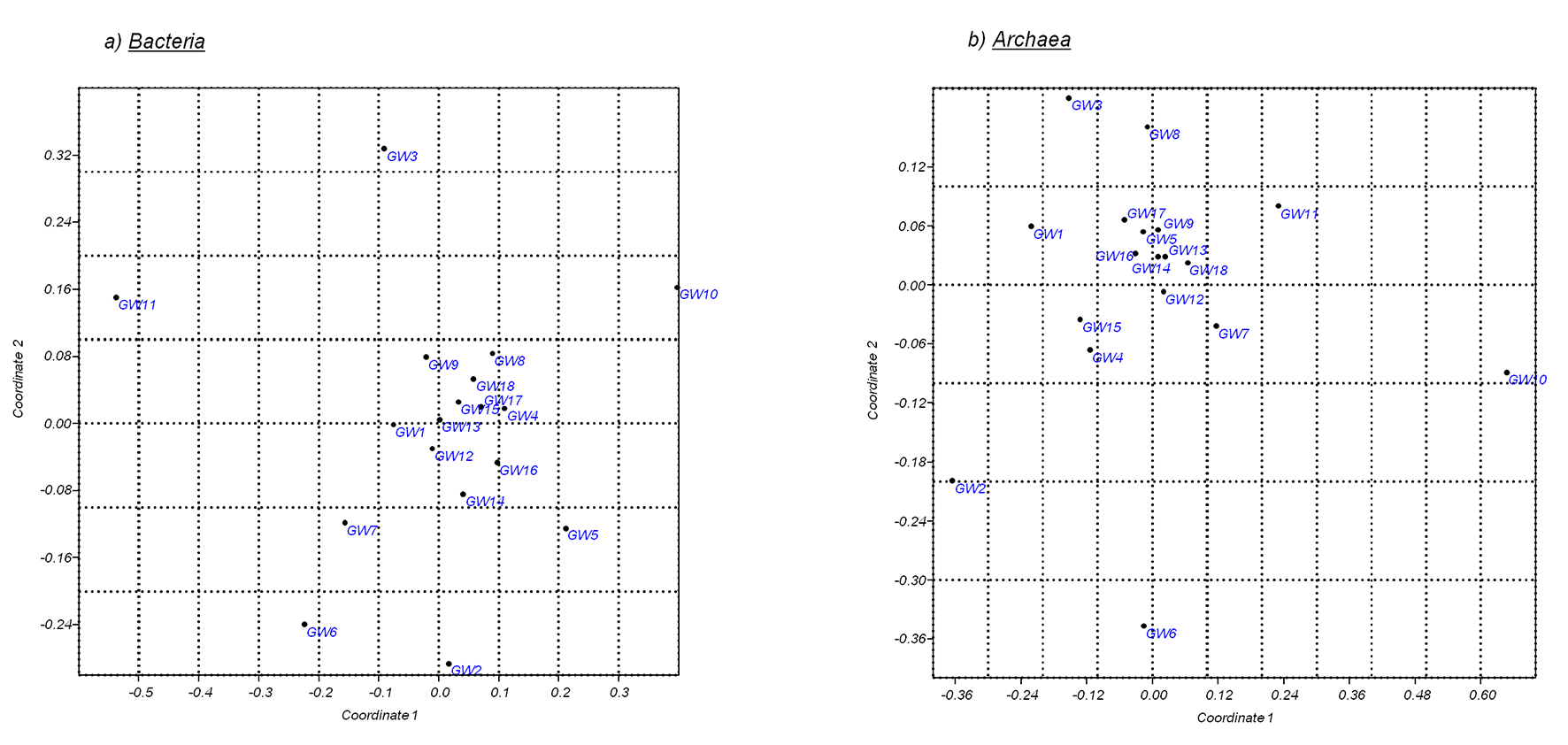

Supplement: Supplementary Figure 4 — Non-Metric Multidimensional Scaling of (A) bacterial and (B) archaeal ARISA dataset. [file Image4.TIF]
